# Revisiting the impact of *BRCA1* pathogenic variants on the aggressiveness of prostate cancer

**DOI:** 10.1093/jncics/pkaf118

**Published:** 2025-12-19

**Authors:** Hajime Sasagawa, Shintaro Narita, Koichi Matsuda, Takeo Kosaka, Yukihide Momozawa

**Affiliations:** Laboratory for Genotyping Development, RIKEN Center for Integrative Medical Sciences, Yokohama, Japan; Department of Urology, Akita University Graduate School of Medicine, Akita, Japan; Department of Urology, Akita University Graduate School of Medicine, Akita, Japan; Laboratory of Clinical Genome Sequencing, Department of Computational Biology and Medical Sciences, Graduate School of Frontier Sciences, The University of Tokyo, Tokyo, Japan; Department of Urology, Keio University School of Medicine, Tokyo, Japan; Laboratory for Genotyping Development, RIKEN Center for Integrative Medical Sciences, Yokohama, Japan

## Abstract

*BRCA1* pathogenic variants are associated with a lower risk of developing prostate cancer than *BRCA2*, but aggressiveness remains unclear. Therefore, screening criteria are insufficiently established. Here, we reanalyzed the impact of *BRCA1* pathogenic variants on aggressiveness using 11 300 prostate cancer patients, adjusting for age and area. The proportion of aggressive prostate cancer was higher in *BRCA1* carriers (86.7%) than in noncarriers (61.1%) (odds ratio = 4.87; 95% confidence interval = 1.05 to 22.60). The proportion of high prostate-specific antigen levels was higher in *BRCA1* carriers (66.7%) than in noncarriers (27.9%) (*P* = 7.61 × 10^−3^). *BRCA1* carriers had a worse tendency than noncarriers for T classification (T3–4: *BRCA1*, 36.4%; noncarriers, 23.2%) and Gleason score (GS8–10: *BRCA1*, 53.3%; noncarriers, 31.0%). Moreover, we observed the first case of *BRCA1*-related aggressive prostate cancer showing long-term survival through early detection and multidisciplinary treatment. These results suggest that recommendations for early prostate cancer screening might need to be reconsidered for *BRCA1* carriers.

Genetic factors influence the development and clinical characteristics of prostate cancer. *BRCA2* pathogenic variants increase the risk of prostate cancer development by 3- to 8.5-fold and are associated with aggressiveness and high mortality,^1^ prompting early screening for *BRCA2* carriers.^2^^,^^3^ On the other hand, *BRCA1* pathogenic variants increase the risk of prostate cancer by 1- to 3-fold.^1^ However, the impact on prostate cancer is lower than that of *BRCA2*, and their impact on aggressiveness is also unclear.^1^ In the National Comprehensive Cancer Network guidelines, screening at age 40 is “recommend for *BRCA2* carriers” but “consider for *BRCA1* carriers.”^3^ The European Association of Urology guidelines strongly recommend prostate-specific antigen (PSA) testing at age 40 for *BRCA2* carriers but omit *BRCA1* carriers,^2^ likely due to insufficient evidence of aggressiveness. In our previous analysis of 7636 prostate cancer patients, *BRCA1* pathogenic variants were not significantly associated with prostate cancer development, so we did not conduct an association analysis with clinical characteristics.^4^ However, a case report of prostate cancer with *BRCA1* pathogenic variants shows a drastic clinical course and difficulty in treatment.^5^ Although *BRCA1* pathogenic variants might have a limited contribution to prostate cancer development, they could influence its aggressiveness. It is worth clarifying this point for the appropriate screening that enables early detection and treatment intervention for aggressive prostate cancer.

We reanalyzed associations between carrier status and the clinical characteristics of 11 300 prostate cancer patients from BioBank Japan between 2003 and 2018^6^^,^^7^ using sequencing data from our previous studies.^4^^,^^8^ In addition, we reported a case of aggressive prostate cancer with a *BRCA1* germline pathogenic variant at Keio University Hospital. The study received approval from the ethical committees of the Institute of Medical Sciences, the University of Tokyo, the RIKEN Center for Integrative Medical Sciences, and Keio University Hospital. All participants provided written informed consent.

Variants predicted to be loss-of-function according to SnpEff (version 4.3t)^9^ or registered as pathogenic or likely pathogenic in ClinVar (version 2023-12-25)^10^ were collectively designated pathogenic variants. Noncarriers were defined as those without pathogenic variants in *BRCA1* and *BRCA2*. Patients with clinical characteristics of either stage T3–4, N1, M1, Gleason score (GS) ≥8, or PSA >20 ng/mL were defined as aggressive prostate cancer. Nonaggressive prostate cancer was defined as T0–2, N0, M0, GS ≤7, and PSA ≤20 ng/mL.

To assess the association between pathogenic variants and clinical characteristics, we used the Mann-Whitney U test for continuous variables and logistic regression models adjusted for age and hospital location for discrete variables. All statistical tests were 2-sided, and statistical significance was set at *P* < .05. All statistical analyses were performed using R, version 4.2.3 (R Foundation for Statistical Computing, Vienna, Austria).

The characteristics of 11 300 prostate cancer patients in BioBank Japan are shown in Table S1. The numbers of *BRCA1* carriers, *BRCA2* carriers, and noncarriers were 19 (0.17%), 120 (1.06%), and 11 161 (98.77%), respectively. The median age at diagnosis (interquartile range) was 69.0 (62.5-74.5) years for *BRCA1* carriers, 69.0 (63.0-74.0) years for *BRCA2* carriers, and 71.0 (65.0-75.0) years for noncarriers. *BRCA1* or *BRCA2* carriers showed a tendency toward younger age at diagnosis than noncarriers.

We evaluated the association between pathogenic variants and the aggressiveness of prostate cancer (Table 1). The proportion of aggressive prostate cancer in *BRCA1* carriers was the same as in *BRCA2* carriers (86.7%), which was higher than in noncarriers (61.1%) (odds ratio [OR] = 4.87; 95% confidence interval [CI] = 1.05 to 22.60; *P* = .043). We investigated each clinical characteristic (Table S2). The proportion of patients with high PSA levels (PSA >20 ng/mL) was significantly higher in *BRCA1* carriers than in noncarriers (*P* = 7.61 × 10^−3^). *BRCA1* carriers had a higher tendency for worse clinical characteristics compared with noncarriers for T classification (T3–4: *BRCA1*, 36.4%; noncarriers, 23.2%) and Gleason score (GS 8–10: *BRCA1*, 53.3%; noncarriers, 31.0%). Although these results were not statistically significant due to insufficient statistical power, they showed a comparable tendency to those of *BRCA2* carriers (T3–4: *BRCA2*, 45.7%; GS 8–10: *BRCA2*, 52.7%).

**Table 1.** The association with *BRCA1/2* pathogenic variants for aggressive and nonaggressive prostate cancer.

	Nonaggressive T0–2 and N0 and M0 and GS ≤7 and PSA ≤20		Aggressive T3–4 or N1 or M1 or GS 8–10 or PSA >20		P^a^	OR (95% CI)^a^
**Gene**	**Number of pathogenic variant carriers**	**Proportion (%)**	**Number of pathogenic variant carriers**	**Proportion (%)**		
*BRCA1*	2	13.33	13	86.67	.043	4.87 (1.05 to 22.60)
*BRCA2*	12	13.33	78	86.67	6.34 × 10^-7^	4.89 (2.62 to 9.14)
Noncarrier	2871	38.88	4514	61.12		

a A logistic-regression model with adjustment for age at diagnosis and hospital location was used.

Abbreviations: GS = Gleason score; PSA = prostate-specific antigen; OR = odds ratio; CI = confidence interval.

We described a case of aggressive prostate cancer with a *BRCA1* pathogenic variant (p.Met1411Thr registered as likely pathogenic in ClinVar) who was treated with multidisciplinary therapy and survived for a long time. The patient visited the Keio University Hospital with hematospermia and was diagnosed with metastatic prostate cancer (stage T3b, N0, M1b; GS, 4 + 4; PSA, 18.57 ng/mL) at age 43. He underwent multidisciplinary treatment, including prostatectomy, hormone therapy, chemotherapy, poly (adenosine diphosphate [ADP]-ribose) polymerase inhibitors, and radiation therapy (Figure 1). Despite the aggressive case, it has been presumed to have led to survival reaching approximately 10 years due to early treatment intervention and multidisciplinary treatment. Although this is a case report, early detection and treatment intervention may have the potential to lead to favorable clinical outcomes for aggressive prostate cancer patients with a *BRCA1* pathogenic variant. Details are described in the Supplementary Material.

**Figure 1.**
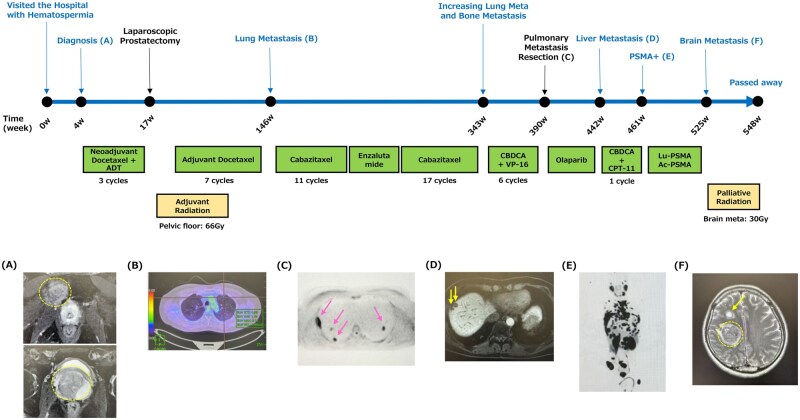
The clinical course of the prostate cancer patient with a *BRCA1* germline pathogenic variant at Keio University Hospital. The clinical course is provided. **A)** MRI of prostate cancer (surrounded by a yellow dotted line) at diagnosis. **B)** CT showing lung metastasis after adjuvant therapy. **C)** PET/CT before pulmonary metastasis resection. The pink arrows indicate the metastatic tumor. **D)** MRI showing liver metastasis (yellow arrow) after treatment with olaparib. **E)** PSMA-PET after treatment with CBDCA and CPT-11. **F)** MRI of brain metastasis after PSMA therapy. It is indicated by a yellow arrow and surrounded by a yellow dotted line. Abbreviations: MRI = magnetic resonance imaging; CT = computed tomography; PET/CT = positron emission tomography/computed tomography; PSMA-PET = prostate-specific membrane antigen positron emission tomography; CBDCA = carboplatin; CPT-11 = irinotecan; ADT = androgen-deprivation therapy; VP-16 = etoposide.

In this study, *BRCA1* carriers were significantly associated with aggressive prostate cancer, and each category tended to be worse than noncarriers, suggesting a similar trend to *BRCA2* carriers. Some studies suggested that *BRCA1* pathogenic variants tend to be associated with aggressive prostate cancer (OR = 1.2–2.0); however, the lower carrier frequency of *BRCA1* (0.2%) compared with *BRCA2* (1.1%) limits the statistical power. Because the aggressiveness of prostate cancer leads to high mortality, clinical management of *BRCA1* carriers should be carefully considered, and larger studies are required.

The disease risk of pathogenic variants does not always correlate with its aggressiveness.^11^^,^^12^ For example, pathogenic variants in *HOXB13* raise prostate cancer risk 2- to 6-fold but are not associated with its aggressiveness.^1^  *HOXB13* interacts with the androgen receptor, a growth and differentiation regulator in prostate biology, contributing to epigenetic reprogramming in tumorigenesis,^13^^,^^14^ which may affect early cellular changes and carcinogenesis. On the other hand, *BRCA1* interacts with various genes and is involved in cellular functions. *MRE11A*, which has been suggested to be correlated with the progression and poor survival of prostate cancer, interacts with *ATM*, *NBN,* and *BRCA1.*^12^^,^^15^ Therefore, the loss-of-function in *BRCA1* may affect *MRE11A*, affecting its progression rather than its development. Further studies on these mechanisms may help elucidate the factors contributing to aggressiveness.

This study has some limitations. We reanalyzed the data from 11 300 prostate cancer patients and found a higher proportion of aggressive prostate cancer in *BRCA1* carriers than in noncarriers. The low number of *BRCA1* carriers is a limitation of this study. However, one of the strengths of this study is that it used samples collected before genetic testing became covered by national insurance, making it most likely that the participants were unaware of their genetic information. In contrast, most previous studies have selected and followed *BRCA1* carriers.^16^^,^^17^ This study is significant because it focused on a nonselected population; as a result, it reduces the potential for bias regarding differences in the recognition of the risk between carriers and noncarriers. However, these results should be carefully interpreted. We also reported a case suggesting the importance of early detection and treatment intervention; however, this is based on only one case. Accumulating more data is indispensable for developing more effective screening criteria for *BRCA1* carriers.

We observed that *BRCA1* pathogenic variants affect prostate cancer aggressiveness such as *BRCA2* pathogenic variants, and the first case of aggressive prostate cancer with a *BRCA1* pathogenic variant had long-term survival through early detection and multidisciplinary treatment. Although further studies and accumulating cases are required, these results suggest that recommendations for early screening for prostate cancer might need to be reconsidered for *BRCA1* carriers.

## Supplementary Material

pkaf118_Supplementary_Data

## Data Availability

The sequence data that support the findings of this study have already been deposited in the NDBC human database (NBDC Research ID: hum0014). The data are available under NBDC Data Sharing Policy (controlled-access data Type-1), and the accession ID is JGAS000203.
