# Supplementary material for: Revisiting the impact of BRCA1 pathogenic variants on the aggressiveness of prostate cancer
Source: JNCI Cancer Spectr. 2025 Dec 19;10(1):pkaf118. doi: 10.1093/jncics/pkaf118 (PMC12803783; doi:10.1093/jncics/pkaf118)
Supplement: pkaf118_Supplementary_Data [file pkaf118_supplementary_data.pdf]

Supplementary Materials

Case Presentation

A 43-year-old man presented with a chief complaint of hematospermia and was diagnosed with metastatic prostate adenocarcinoma (**Figure 1A**) (Gleason score 4 + 4) with an elevated prostate-specific antigen (PSA) level of 18.6 ng/mL, clinical stages T3b, N0, and M1b followed by neoadjuvant androgen deprivation and docetaxel therapy, prostatectomy, and metastatectomy of the pubic bone. Pathological examination revealed residual adenocarcinoma of the prostate and pubic bone (pathological stage T3b; positive surgical margins). After surgery, the patient received adjuvant radiation therapy (66 Gy) on the pelvic floor and docetaxel. Approximately 2 years after the initial diagnosis, lung metastasis appeared (**Figure 1B**), and cabazitaxel was administered. The patient was followed up with intermittent cabazitaxel administration. The course up to this point has been previously reported in a case showing that multidisciplinary treatment and liquid biopsy are helpful for metastatic prostate cancer at a young age [1].

The patient's quality of life was maintained without disease progression by the appropriate and intermittent use of cabazitaxel and enzalutamide. Approximately 6 years after surgery, lung metastasis showed a tendency to increase again, and bone metastasis appeared. Because the PSA level was low (0.03 ng/mL), a needle biopsy was performed on the lung metastasis for pathological tissue evaluation. The pathological diagnosis was prostate cancer with neuroendocrine transformation, and chemotherapy with a combination of carboplatin and etoposide was administered. Pulmonary metastasis resection was performed for the metastatic lesions that showed a tendency to grow (**Figure 1C**). Gene panel testing was performed on a needle biopsy specimen from the metastatic lesion, and the *BRCA1* variant was detected. The patient received genetic counseling and underwent germline *BRCA1* testing, which revealed a

germline variant (p.Met1411Thr registered as likely pathogenic in ClinVar). Olaparib, a poly (ADP [adenosine diphosphate]-ribose) polymerase (PARP) inhibitor, has been administered. The patient's PSA level was low (0.09 ng/ml). Five months after starting olaparib, the lung and bone metastases worsened, and liver metastases appeared (**Figure 1D**). The patient was diagnosed with progressive disease by imaging; however, the PSA level remained low (0.22 ng/mL). Combined carboplatin and irinotecan therapy was introduced, but continued treatment was difficult owing to hematologic toxicity of grade 3 and a greater decrease in hemoglobin and platelet count. Therefore, although it was not approved in Japan, the patient and his family requested that they travel to a country where it was approved and undergo positron emission tomography with a prostate-specific membrane antigen (PSMA) (**Figure 1E**). The PSA level was also low (1.18 ng/mL). Because positive findings were observed for PSMA in metastatic lesions, Lu-PSMA and Ac-PSMA therapies were continued in the country where the patient traveled. Six months after starting PSMA therapy, the patient achieved a partial response on imaging, and the quality of life was maintained except for decreased salivary gland secretion and taste disorders. Brain metastases and lower limb paralysis appeared approximately 15 months after the introduction of PSMA (**Figure 1F**). Palliative radiation therapy was administered to brain metastases (30 Gy); however, the patient's performance status rapidly declined, making aggressive follow-up treatment difficult, and the best supportive care was selected. The patient died approximately 10 years and 6 months after the initial diagnosis, 6 months following the appearance of brain metastases.

In addition to the removal of the primary lesion and simultaneous surgery of the bone metastases at the time of initial diagnosis and multidisciplinary treatment with neoadjuvant and adjuvant drugs during the perioperative period, the patient's PSA level was low. At the time of recurrence, platinum treatment was selected for dedifferentiated cancer diagnosed by a

pathological diagnosis of the metastatic lesion, and multidisciplinary treatment such as PARP inhibitors and PSMA treatment based on gene panel diagnosis was continuously performed. Thus, it was presumed to have led to long-term survival despite aggressive prostate cancer with *BRCA1* germline pathogenic variants.

References

[1] Kosaka, T., Hongo, H., Oya, M. Complete response with early introduction of cabazitaxel in a patient with multiple lung metastases of castration-resistant prostate cancer following the early detection of metastases using liquid biopsy: a case report. BMC Cancer. 2019;19:562.

Table S1. Characteristics of patients

		Patients<br>N = 11,300
Age at entry, median (IQR)		73.0 (68.0-77.0)
Age at diagnosis, median (IQR)		71.0 (65.0-75.0)
TNM classification: T (%)		
	T4	284 (2.5)
	T3	1,396 (12.4)
	T2	3,440 (30.4)
	T1	2,028 (18.0)
	T0	14 (0.1)
	Unknown	4,138 (36.6)
TNM classification: N (%)		
	N1	443 (3.9)
	N0	6,619 (58.6)
	Unknown	4,238 (37.5)
TNM classification: M (%)		
	M1	520 (4.6)
	M0	6,444 (57.0)
	Unknown	4,336 (38.4)
Gleason Score (%)		
	8-10	2,921 (25.8)
	≤ 7	6,416 (56.8)
	Unknown	1,963 (17.4)
Maximum PSA before treatment, ng/ml		
	>20	2,254 (20.0)
	10< - ≤20	1,932 (17.1)
	4< - ≤10	3,595 (31.8)
	≤4	228 (2.0)
	Unknown	3,291 (29.1)

Abbreviations: IQR = interquartile range, PSA = prostate-specific antigen.

Table S2. Clinical characteristics of prostate cancer patients with *BRCA1/2* pathogenic variants compared with non-carriers

Gene	T0-2		T3-4		P-value[a]	OR (95% CI)[a]
	Number	Proportion (%)	Number	Proportion (%)		
BRCA1	7	63.64	4	36.36	0.205	2.26 (0.64-7.95)
BRCA2	38	54.29	32	45.71	8.81×10^{-6}	3.05 (1.86-4.98)
Non-Carrier	5,437	76.78	1,644	23.22		

Gene	N0		N1		P-value	OR (95% CI)
	Number	Proportion (%)	Number	Proportion (%)		
BRCA1	10	90.91	1	9.09	0.661	1.59 (0.20-12.72)
BRCA2	61	83.56	12	16.44	2.52×10^{-4}	3.28 (1.74-6.20)
Non-Carrier	6,548	93.84	430	6.16		

Gene	M0		M1		P-value	OR (95% CI)
	Number	Proportion (%)	Number	Proportion (%)		
BRCA1	10	90.91	1	9.09	0.796	1.32 (0.16-10.55)
BRCA2	60	82.19	13	17.81	4.85×10^{-4}	3.00 (1.62-5.57)
Non-Carrier	6,374	92.65	506	7.35		

Gene	GS ≤7		GS 8-10		P-value	OR (95% CI)
	Number	Proportion (%)	Number	Proportion (%)		
BRCA1	7	46.67	8	53.33	0.062	2.66 (0.95-7.44)
BRCA2	44	47.31	49	52.69	3.28×10^{-6}	2.71 (1.78-4.13)
Non-Carrier	6,365	68.97	2,864	31.03		

Gene	PSA ≤20		PSA >20		P-value	OR (95% CI)
	Number	Proportion (%)	Number	Proportion (%)		
BRCA1	4	33.33	8	66.67	7.61×10^{-3}	5.50 (1.57-19.25)
BRCA2	38	48.72	40	51.28	4.68×10^{-6}	2.98 (1.87-4.76)
Non-Carrier	5,713	72.14	2,206	27.86		

Abbreviations: GS = Gleason score, PSA = prostate-specific antigen, OR = odds ratio, CI = confidence interval

[a] A logistic-regression model with adjustment for age at diagnosis and hospital location was used.
